# Cardiovascular outcomes associated with SGLT2 inhibitor therapy in patients with type 2 diabetes mellitus and cancer: a systematic review and meta-analysis

**DOI:** 10.1186/s13098-024-01354-4

**Published:** 2024-05-22

**Authors:** Hsiao-Huai Kuo, Kuang-Te Wang, Hsin-Hao Chen, Zih-Yin Lai, Po-Lin Lin, Yung-Jen Chuang, Lawrence Yu-Min Liu

**Affiliations:** 1 Department of Biomedical Engineering and Environmental Sciences, National Tsing Hua University, Hsinchu, Taiwan; 2 Department of Pharmacy, Hsinchu Municipal MacKay Children’s Hospital, Hsinchu, Taiwan; 3 Department of Pharmacy, Hsinchu MacKay Memorial Hospital, Hsinchu, Taiwan; 4 Department of Nursing, Hsin Sheng Junior College of Medical Care and Management, Taoyuan, Taiwan; 5 Division of Cardiology, Department of Internal Medicine, Taitung MacKay Memorial Hospital, Taitung, Taiwan; 6 Department of Medicine, Mackay Medical College, New Taipei, Taiwan; 7 Nursing, and Management, MacKay Junior College of Medicine, Taipei, Taiwan; 8 Department of Family Medicine, Hsinchu MacKay Memorial Hospital, Hsinchu, Taiwan; 9 School of Medicine, National Tsing Hua University, Hsinchu, Taiwan; 10 Institute of Bioinformatics and Structural Biology, National Tsing Hua University, Hsinchu, Taiwan; 11 Division of Cardiology, Department of Medicine, Hsinchu MacKay Memorial Hospital, Hsinchu, Taiwan

**Keywords:** SGLT2 inhibitors, Cardiovascular outcomes, Cancer, Cardiotoxicity, Meta-analysis

## Abstract

**Background:**

Cancer patients with diabetes are at increased risk for cardiovascular diseases due to common risk factors and well-documented drug-associated cardiotoxicity. Sodium-glucose cotransporter-2 (SGLT2) inhibitors have shown cardiovascular benefits in patients with diabetes, but their effects on cancer patients remain unclear. This study aimed to evaluate the cardiovascular outcomes associated with SGLT2 inhibitor therapy in patients with concomitant diabetes and cancer.

**Methods:**

We conducted a systematic review and meta-analysis of cohort studies comparing cardiovascular outcomes between cancer patients with diabetes receiving SGLT2 inhibitors and those not receiving SGLT2 inhibitors. PubMed, Embase, and the Cochrane Library were searched from inception to February 29, 2024. The primary outcome was all-cause mortality, and the secondary outcomes were heart failure hospitalization, and adverse events. Random-effect models were used to calculate pooled risk ratios (RR) with 95% confidence intervals (CI). Subgroup and sensitivity analyses were conducted to identify potential sources of heterogeneity and explore the effect of SGLT2 inhibitors on mitigating cardiotoxicity.

**Results:**

Nine cohort studies involving 82,654 patients were included. SGLT2 inhibitor use was associated with a significantly lower risk of all-cause mortality (RR 0.46, 95% CI 0.31–0.68, *P* < 0.0001; I^2^ = 98%) and heart failure hospitalization (RR 0.49, 95% CI 0.30–0.81, *P* = 0.006; I^2^ = 21%) compared to non-use. The mortality benefit remained significant in patients receiving anthracycline chemotherapy (RR 0.50, 95% CI 0.28–0.89, *P* = 0.02; I^2^ = 71%). SGLT2 inhibitor use was also associated with a lower risk of sepsis (RR 0.32, 95% CI 0.23–0.44, *P* < 0.00001; I^2^ = 0%) and no increased risk of diabetic ketoacidosis (RR 0.66, 95% CI 0.20–2.16, *P* = 0.49; I^2^ = 0%).

**Conclusions:**

SGLT2 inhibitor therapy is associated with lower risks of all-cause mortality and heart failure hospitalization in patients with concomitant diabetes and cancer. These findings suggest that SGLT2 inhibitors may offer cardiovascular benefits in this high-risk population. Randomized controlled trials are needed to validate these findings and evaluate the safety and efficacy of SGLT2 inhibitors in specific cancer types and treatment regimens.

**Supplementary Information:**

The online version contains supplementary material available at 10.1186/s13098-024-01354-4.

## Introduction

Cancer treatment has made remarkable progress, resulting in a growing number of cancer survivors [1]. However, cancer patients often share risk factors, such as smoking and diabetes, which increase their likelihood of experiencing cardiovascular events and reduce cancer survival [2–4]. Diabetes has also been shown to impede the effectiveness of certain anti-cancer drugs [5, 6]. As the population of cancer survivors expands, there is an increasing need for strategies to address the complex relationship between cancer and cardiovascular health.

Patients who have undergone cancer treatment often face cardiovascular complications, particularly chemotherapy-associated heart failure, adding complexity to their long-term health management [7]. Notably, anthracyclines are well known for their potential to cause cardiotoxicity. In addition, the combination of chemotherapy with radiotherapy or targeted therapy has been frequently reported to contribute to cardiovascular complications [8, 9].

Early diagnosis of cardiac dysfunction and prompt initiation of neurohormonal therapy have been associated with greater left ventricular ejection fraction (LVEF) recovery [10]; however, their effectiveness remains suboptimal [11, 12]. Novel therapies, including angiotensin receptor-neprilysin inhibitors (ARNI), therapies targeting apoptosis or oxidative stress, and stem cell therapy, have also been actively explored [13, 14].

Sodium-glucose co-transporter 2 (SGLT2) inhibitors are a novel class of oral diabetes drugs that block glucose reabsorption via SGLT2 in the proximal tubules of the kidneys, leading to increased glucose excretion in the urine. These drugs have demonstrated significant cardiovascular benefits, particularly in preventing heart failure in both diabetes and non-diabetes patients [15]. Recent cohort studies have suggested possible cardiovascular benefits of SGLT2 inhibitors in cancer patients [16], and animal studies and laboratory investigations have proposed mechanisms underlying this cardioprotective effect [17–19].

Despite these findings, the potential role of SGLT2 inhibitors in mitigating cardiotoxicity and protecting the cardiovascular health of cancer survivors exposed to chemotherapy remains unclear. We thus performed a comprehensive systematic review and meta-analysis of available cohort studies to evaluate the cardiovascular effects of SGLT2 inhibitors in this population. Our aim is to synthesize the available observational data and generate hypotheses for novel treatment strategies. The findings from this study may provide a foundation for future clinical trials investigating the role of SGLT2 inhibitors in protecting the cardiovascular health of cancer survivors.

## Methods

### Search strategy and selection criteria

The review protocol has been registered in the PROSPERO International Prospective Register of Systematic Reviews (CRD 42,023,487,280) and was written according to the Preferred Reporting Items for Systematic Reviews and Meta-Analyses (PRISMA) 2020 statement [20].

Two reviewers (H.H.K. and H.H.C.) conducted a comprehensive search of multiple electronic databases, including PubMed, Embase (excluding Medline), and the Cochrane Library, from inception to February 29, 2024, to identify relevant retrospective cohort studies investigating the association between SGLT2 inhibitors and cardiovascular outcomes in cancer patients receiving cancer therapies. The search strategy included combinations of keywords and MeSH terms related to SGLT2 inhibitors, anthracyclines, cancer, neoplasms, malignancy, cardiotoxicity, and cardiovascular outcomes.

Studies were considered eligible if they met the following criteria: [1] retrospective or prospective cohort studies; [2] included patients with active cancer undergoing cardiotoxic therapies such as chemotherapy, immune therapy, or radiotherapy; [3] compared cardiovascular outcomes between patients receiving SGLT2 inhibitors and those not receiving SGLT2 inhibitors; and [4] reported data on at least one of the following outcomes: all-cause mortality, heart failure hospitalizations, or adverse events. Studies focusing on specific cancer types or SGLT2 inhibitor agents were also eligible. Two reviewers (H.H.K and H.H.C) independently screened the titles and abstracts and reviewed the full texts of potentially eligible studies. Disagreements were resolved by discussion with a third reviewer (L.Y.L.). Reference lists of the included studies were also manually searched for additional relevant articles.

### Data extraction and quality assessment

Two reviewers (H.H.K. and H.H.C.) independently extracted data from the included studies using a pre-designed form. The extracted information included the first author, publication year, country, study period, study type, number of patients, mean age, type of cancer and chemotherapy, study outcomes, and average follow-up years. Any discrepancies were resolved by discussion with a third reviewer (L.Y.L.). The methodological quality of the included cohort studies was assessed using the Newcastle-Ottawa Scale (NOS). This scale evaluates three domains: selection of study groups, comparability of groups, and ascertainment of outcomes. Studies with scores ≥ 7 were considered to be of high quality. The quality assessment was performed independently by two reviewers (H.H.K. and H.H.C.), with disagreements resolved by consensus.

### Statistical analysis and data synthesis

The primary outcome was all-cause mortality, and the secondary outcomes were heart failure hospitalization and adverse events such as sepsis and diabetic ketoacidosis (DKA). The effects of SGLT2 inhibitors on the outcomes were assessed by pooled risk ratio (RR) with 95% confidence intervals (CI). We used the random-effect method to pool the results from the included studies, accounting for variance among the studies. Heterogeneity was assessed using the Cochran’s Q test and quantified by the I^2^ statistic. I^2^ values of 25%, 50%, and 75% were considered to represent low, moderate, and high heterogeneity, respectively. To address heterogeneity, we conducted subgroup and sensitivity analyses. Subgroup analyses were performed based on the use of anthracyclines and the type of cancer, if available. In the sensitivity analyses, we omitted one study at a time to evaluate the robustness of the results.

Publication bias was assessed using funnel plots. A two-tailed *P* < 0.05 was considered statistically significant. All analyses were performed using the Review Manager (version 5.4.1).

## Results

### Study selection and characteristics

**Fig. 1:**
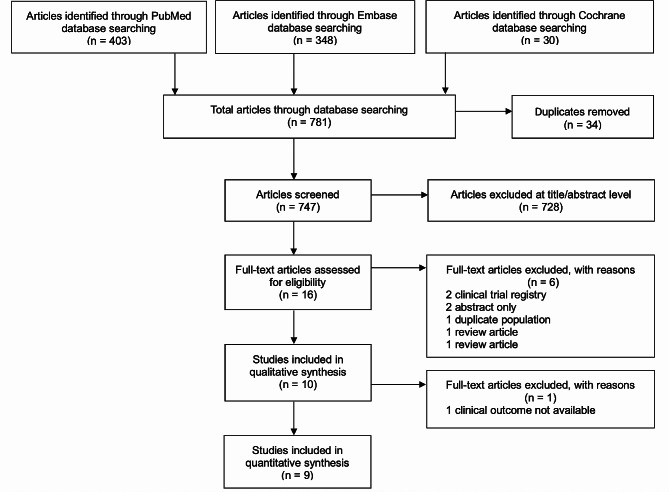
Flow chart of the literature search and study selection process

The initial literature search identified 781 articles. After removing 34 duplicates and screening titles and abstracts, 16 full-text articles were assessed for eligibility. Of these, six articles were excluded because they were not original studies or contained duplicate populations. One study did not report primary outcome data, and nine studies involving a total of 82,654 patients were included in the quantitative meta-analysis (Fig. 1).

Table 1 summarizes the characteristics of the included studies. Four studies were conducted in North America (the United States and Canada) [21–24], three in Asia (Taiwan and South Korea) [25–27], one in Israel [28], and one using a global database [29]. Study periods ranged from January 2010 to August 2022. The total number of patients in the SGLT2 inhibitor group was 19,831 compared to 62,823 in the control group. The average age ranged from 60 to 74.8 years old. Where reported, chemotherapy regimens included anthracyclines, alkylating agents, antimetabolites, platinum-based therapies, tyrosine kinase inhibitors, and immune checkpoint inhibitors. Three studies included only cancer patients receiving anthracyclines chemotherapy [21, 24, 27]. Seven of these studies included various cancer types, while the other two consisted of a single type of cancer each: non-small cell lung cancer and hepatoma, respectively [22, 23]. Average follow-up durations ranged from 1.5 to 4.8 years. Study quality assessment using the NOS indicated that all studies were of moderate to high quality (NOS score ≥ 6).

**Table 1:** Characteristics of the included studies

First author, year	Country	Study period	Study type	Total No. of patients (SGLT2i/ control)	Average Age	SGLT2i	Types of cancer	Chemotherapy type	Study Outcomes	Average follow-up year	NOS
Perelman, 2024 [28]	Israel	November 2015 to August 2022	Retrospective cohort	119 (24/95)	71	Empagliflzin (83%), dapagliflozin (17%)	Various, including NSCLC, RCC, hepatoma, and others	Immune checkpoint inhibitors	All-cause mortality, MACE, arrhythmia	2.3	6
Huang, 2024 [26]	Taiwan	January 2016 to December 2018	Retrospective propensity score-matched cohort	50,133 (16,711/33,422)	62	Not specified	Various, including colorectal, breast, head and neck, and others	Not specified	Cancer-specific mortality, all-cause mortality	4.5/ 4.8	9
Hwang, 2023 [27]	South Korea	January 2014 to December 2021	Retrospective propensity score-matched cohort	3116 (779/2337)	Not reported	Not specified	Various, including lymphoma, breast, genitourinary, and others	Anthracyclines (100%), alkylating agents, antimicrobial agents, HER2 inhibitors, VEGF-targeting agents	All-cause mortality, HHF, AMI, ischemic stroke	Not reported	7
Abdel-Qadir, 2023 [24]	Canada	January 2016 to December 2019	Retrospective propensity score-matched cohort	933 (99/834)	71	Dapagliflozin, empagliflozin, canagliflozin	Various, including breast, lymphoma, gastric, and others	Anthracyclines	HHF, HF incident, CVD hospitalization, all-cause death, hypoglycemia, DKA	1.5	9
Luo, 2023 [23]	USA	2014 to 2017	Retrospective cohort	24,915 (531/24,384)	72.5	canagliflozin, dapagli- flozin, empagliflozin and ertugliflozin	Non-small cell lung cancer	Not specified	All-cause mortality	1.5	9
Chiang, 2023 [25]	Taiwan	January 2010 to December 2021	Retrospective propensity score-matched cohort	1756 (878/878)	65	Empagliflozin (49%), dapagliflozin (38%), canagliflozin (14%)	Various, including gastrointestinal, genitourinary, thoracic, and others	Various, including antimetabolites, platinum, plant alkaloids, anthracyclines, alkylating agents and others	All-cause mortality, HHF, DKA, urosepsis, sepsis, hypogly- caemia, AKI and Fournier’s gangrene	1.6	9
Avula, 2023 [29]	Global	January 2013 to April 2020	Retrospective propensity score-matched cohort	1280 (640/640)	67.6	Dapagliflozin, empagliflozin, canagliflozin	Various, including hematologic, gastrointestinal, breast, and others	Various, including anthracyclines, alkylating agents, antimetabolites, monoclonal antibodies, small-molecule tyrosine kinase inhibitors, proteasome inhibitors, radiotherapy, and others	All-cause mortality, HF exacerbation	2	9
Gongora, 2022 [21]	USA	Before September 2020	Retrospective propensity score-matched cohort	128 (32/96)	60	Empagliflozin (50%), canagliflozin (34%), dapagliflozin (16%)	Various, including lymphoma, breast, genitourinary, and others	anthracyclines	HF incidence, HF admissions, development of CM or clinically significant arrhythmia, all-cause mortality, sepsis, neutropenic fever	1.5	8
Hendryx, 2022 [22]	USA	2014 to 2019	Retrospective propensity score-matched cohort	274 (137/137)	74.8	canagliflozin, dapagliflozin, empagliflozin, and ertugliflozin	Hepatocellular carcinoma	Not specified	All-cause mortality	1.7	9

AKI: acute kidney injury; AMI: acute myocardial infarction; CM: cardiomyopathy; CVD: cardiovascular disease; DKA: diabetic ketoacidosis; HER, human epidermal growth factor receptor; HF: heart failure; HHF: heart failure hospitalization; MACE: major adverse cardiovascular event; NSCLC: non-small cell lung cancer; RCC: renal cell carcinoma, UTI: urinary tract infection; VEGF, vascular endothelial growth factor

### Meta-analysis of mortality

**Fig. 2:**
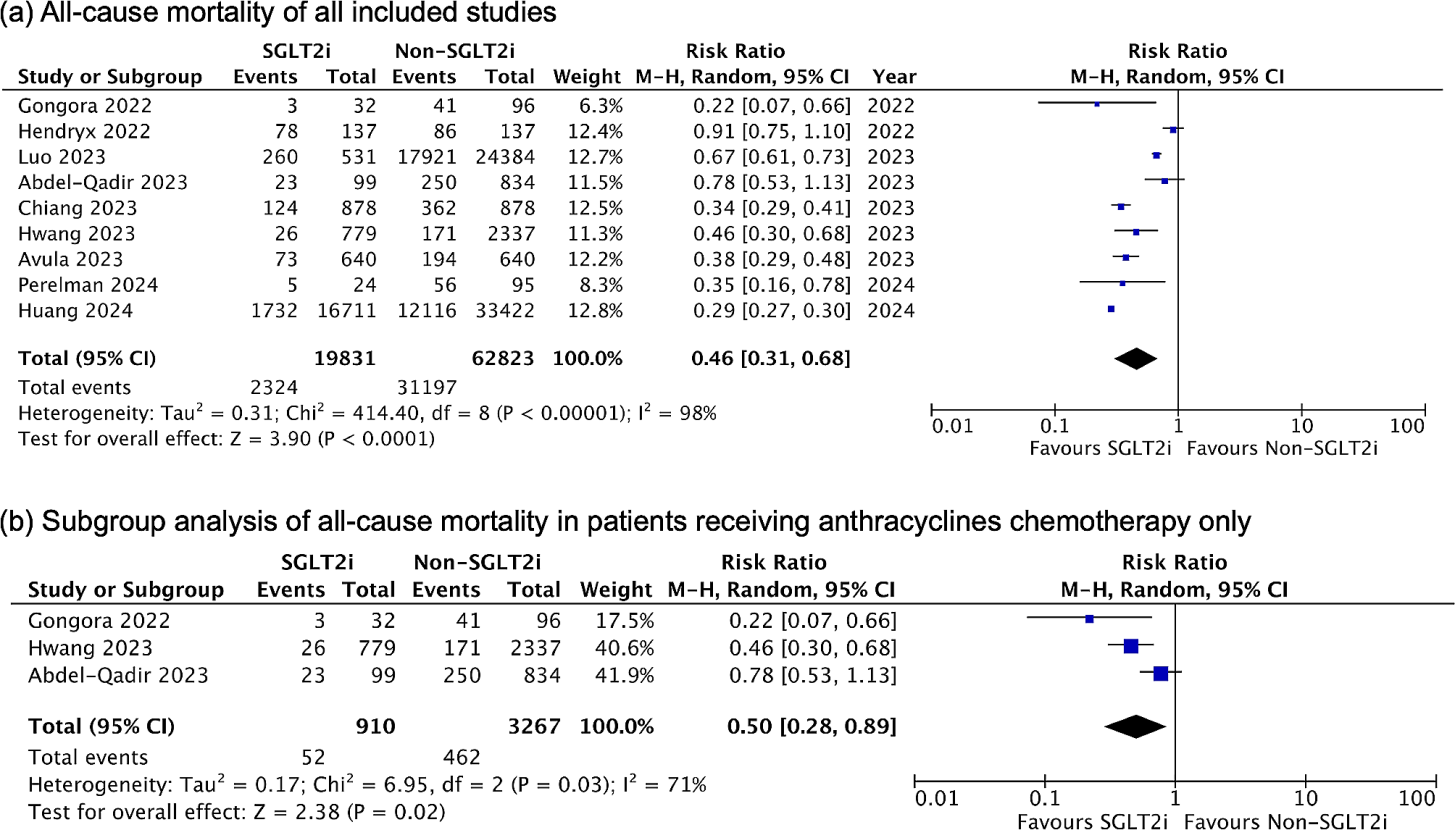
Forest plots of all-cause mortality. (**a**) All included studies (**b**) Subgroup analysis of patients receiving anthracyclines chemotherapy only

All nine studies included in the quantitative meta-analysis reported all-cause mortality as an outcome. As shown in Fig. 2a, SGLT2 inhibitor use was associated with a significantly lower risk of mortality compared to non-use (RR 0.46, 95% CI 0.31–0.68, *P* < 0.0001). However, high heterogeneity between studies was found in this pooled result (I^2^ = 98%). Subgroup analysis restricted to studies with anthracyclines chemotherapy found the mortality benefit remained significant (RR 0.50, 95% CI 0.28–0.89, *P* = 0.02) with moderate heterogeneity (I^2^ = 71%) (Fig. 2b). The all-cause mortality benefit was seen in breast cancer and lung cancer, but it was not statistically significant in hepatoma (Supplementary Fig. 1). We also conducted a sensitivity analysis by sequentially excluding one study at a time to evaluate the robustness of the results, and the pooled results remained consistent.

### Meta-analysis of heart failure hospitalization

**Fig. 3:**
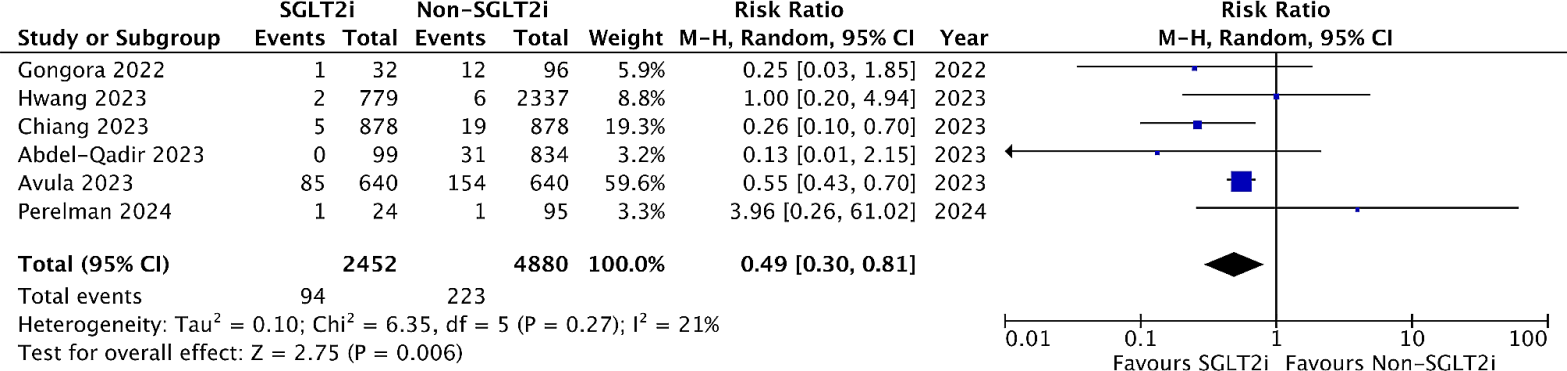
Forest plot of heart failure hospitalization

Six studies reported hospitalization due to heart failure. Patients who used SGLT2 inhibitors had a significantly lower risk for heart failure hospitalization than those without SGLT2 inhibitors (RR 0.49, 95% CI 0.30–0.81, *P* = 0.006). The observed heterogeneity was low (I^2^ = 21%) (Fig. 3). Subgroup analysis restricted to studies with anthracyclines chemotherapy showed a trend for improvement in heart failure hospitalization with low heterogeneity (RR 0.44, 95% CI 0.13–1.5, *P* = 0.19; I^2^ = 12%). One study enrolled cancer patients with prior diagnosis of cancer therapy-related cardiomyopathy or heart failure [29]. By omitting such a study in the sensitivity analysis, the risk ratio of heart failure hospitalization remained similar at 0.44 but became statistically insignificant (RR 0.44, 95% CI 0.17–1.13, *P* = 0.09).

### Meta-analysis of adverse events

**Fig. 4:**
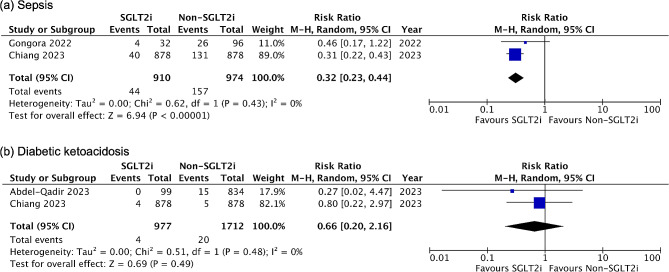
Forest plots of the adverse events. (**a**) Sepsis (**b**) Diabetic ketoacidosis

Five of the nine studies reported various adverse events associated with SGLT2 inhibitor use, including DKA, sepsis, urinary tract infection, genital infection, arrhythmia, hypoglycemia, amputation, and fournier’s gangrene. The pooled estimate for sepsis from two studies revealed a significantly lower risk in the SGLT2 inhibitor group than the non-SGLT2 inhibitor group (RR 0.32, 95% CI 0.23–0.44, *P* < 0.00001) (Fig. 4a). Moreover, the risk of DKA was not increased with SGLT2 inhibitor use in the pooled data (RR 0.66, 95% CI 0.20–2.16, *P* = 0.49) (Fig. 4b).

### Publication bias

The funnel plot of mortality estimates appeared asymmetric, suggesting possible publication bias (Supplementary Fig. 2). However, sensitivity analyses using a fixed-effects model showed results consistent with the main random-effects analysis.

## Discussion

This systematic review and meta-analysis of nine retrospective cohort studies provides the most up-to-date evidence that SGLT2 inhibitors have a beneficial effect on mortality and reducing cardiotoxicity in patients with cancer and type 2 diabetes mellitus undergoing cancer therapy. The pooled results involving 82,654 patients demonstrated that SGLT2 inhibitor use was associated with a significant 54% reduction in the risk of all-cause mortality (RR 0.46, 95% CI 0.31–0.68, *P* < 0.0001) and a 51% reduction in heart failure hospitalization (RR 0.49, 95% CI 0.30–0.81, *P* = 0.006) compared to patients not receiving SGLT2 inhibitors. Although the mortality analysis included a broad population of cancer patients, resulting in high heterogeneity (I^2^ = 98%), the significant risk reduction and consistent findings in the subgroup and sensitivity analyses strongly support the hypothesis that SGLT2 inhibitors may improve outcomes in cancer patients. Notably, the mortality benefit remained significant in the subgroup of patients receiving anthracycline chemotherapy (RR 0.50, 95% CI 0.28–0.89, *P* = 0.02) with moderate heterogeneity (I^2^ = 71%). Furthermore, SGLT2 inhibitor use was associated with a lower risk of sepsis (RR 0.32, 95% CI 0.23–0.44, *P* < 0.00001) and no increased risk of diabetic ketoacidosis (RR 0.66, 95% CI 0.20–2.16, *P* = 0.49). These findings suggest that SGLT2 inhibitors have a cardioprotective effect in mitigating anthracycline-induced cardiotoxicity without increasing the risk of serious adverse events.

The individual cohort studies included in this meta-analysis provide further insights into the potential cardioprotective effects of SGLT2 inhibitors in various cancer populations. Gongora et al. reported that SGLT2 inhibitor use was associated with fewer cardiac events, primarily driven by a reduction in heart failure admissions and new diagnoses of cardiomyopathy among patients with diabetes treated with anthracyclines [21]. Similarly, Chiang et al. observed a threefold lower rate of heart failure hospitalization and improved overall survival in SGLT2 inhibitor recipients than in non-recipients in a broader population of cancer patients treated with different types of chemotherapy drugs [25]. The effect of SGLT2 inhibitors on all-cause mortality was also observed in cancer patients treated with immune checkpoint inhibitors [28]. SGLT2 inhibitor use, in addition to guideline-directed medical therapy, reduced heart failure exacerbations, all-cause mortality, and adverse renal outcomes in patients who already had cancer therapy-related cardiac dysfunction or heart failure at baseline [29]. Major adverse cardiovascular events, a composite outcome of heart failure hospitalization, acute myocardial infarction, ischemic stroke, and death, were significantly lower in cancer patients receiving SGLT2 inhibitors [27]. Thus, SGLT2 inhibitors may offer cardiovascular benefits across different cancer types and treatment regimens.

The findings of this meta-analysis are consistent with the results of large clinical trials that have demonstrated the cardiovascular and renal benefits of SGLT2 inhibitors in patients with diabetes. The DECLARE-TIMI 58 trial demonstrated that dapagliflozin reduced the risk of hospitalization for heart failure and cardiovascular death in patients with type 2 diabetes and established cardiovascular disease or multiple risk factors [30]. Similarly, the CANVAS program found that canagliflozin reduced the risk of cardiovascular events and heart failure hospitalizations in patients with type 2 diabetes and high cardiovascular risk [31]. These trials highlight the potential of SGLT2 inhibitors to improve cardiovascular outcomes in patients with diabetes, which may extend to cancer populations with a high prevalence of diabetes and cardiovascular risk factors.

Several mechanisms have been proposed to explain the cardioprotective effects of SGLT2 inhibitors in patients with cancer and diabetes undergoing anthracycline-based chemotherapy. The potential mechanisms underlying this cardioprotection may involve the reduction of oxidative stress, inflammation, and apoptosis, as well as the improvements in mitochondrial function, fatty acid metabolism, and cardiac energy production, as suggested by preclinical studies [32–35]. SGLT2 inhibitors have been shown to increase the utilization of ketone bodies as an alternative fuel source, leading to improved cardiac efficiency and reduced oxidative stress [36]. Additionally, SGLT2 inhibitors may reduce the cardiac workload by promoting natriuresis and reducing preload and afterload [37]. These mechanisms may be particularly relevant in cancer patients, who are at increased risk of cardiovascular complications due to the direct cardiotoxic effects of cancer therapies and the high prevalence of cardiovascular risk factors [38]. Furthermore, SGLT2 inhibitors have been shown to have direct anti-tumor effects in preclinical studies, suggesting a potential dual benefit in patients with cancer [39, 40].

Interestingly, the subgroup analysis in this meta-analysis suggested that the mortality benefit of SGLT2 inhibitors may be more pronounced in patients with breast cancer and lung cancer compared to those with hepatoma. Preclinical studies have shown that SGLT2 inhibitors may have anti-cancer effects on certain tumor types. For example, in vitro studies have demonstrated that SGLT2 inhibitors can reduce the growth and proliferation of breast cancer cells by inhibiting glucose uptake and inducing apoptosis [41]. Similarly, SGLT2 inhibitors have been shown to attenuate the development of lung adenocarcinoma in mouse models by reducing glucose uptake and activating the AMPK pathway [42]. Animal experiments and cohort studies also suggest potential benefits of SGLT2 inhibitors in non-alcoholic fatty liver disease and hepatocellular carcinoma [22, 43, 44]. These findings highlight the need for further research to evaluate the potential anti-cancer effects of SGLT2 inhibitors in specific tumor types and to elucidate the underlying mechanisms.

These findings have significant clinical implications for primary care physicians, who are often involved in the long-term care of these patients and the management of their comorbidities. The results suggest that incorporating SGLT2 inhibitors into diabetes therapy for cancer patients may improve cardiovascular outcomes and overall survival without increasing the risks of DKA and sepsis.

### Strengths and limitations

This meta-analysis included a large sample size of cancer patients from 9 retrospective cohort studies, increasing the power to detect significant associations. The reduction in all-cause mortality and heart failure hospitalization was significant and consistent in subgroup and sensitivity analyses, indicating the robustness of the results.

However, this study has several limitations to consider when interpreting the results. First, all included studies were observational, and thus, residual confounding due to unmeasured or inadequately measured variables cannot be excluded despite the adjustment for potential confounders in the individual studies. Furthermore, the studies relied on administrative claims data or electronic health records, which may lack detailed clinical information or have misclassification of exposures and outcomes. The duration and dosage of SGLT2 inhibitor use varied across studies, and the optimal timing and duration of treatment remain unclear. Second, the high heterogeneity observed in the mortality analysis may be due to differences in patient populations, cancer types, and treatment regimens across studies. Although efforts to account for the heterogeneity through subgroup and sensitivity analyses, the findings should be interpreted with caution. Third, the duration of follow-up varied among the included studies, ranging from 1.5 to 4.8 years, which may not be sufficient to capture long-term safety and efficacy in cancer survivors. Finally, all patients included in the cohort studies had pre-existing diabetes, and whether the benefits of SGLT2 inhibitors can be extrapolated to non-diabetic cancer patients remains to be determined.

## Conclusions

This systematic review and meta-analysis suggests that SGLT2 inhibitor use may be associated with improved cardiovascular outcomes, particularly reduced mortality and heart failure hospitalization, in cancer patients with diabetes receiving cancer therapies. These findings warrant future randomized controlled trials to confirm the cardioprotective effects of SGLT2 inhibitors in cancer patients with and without diabetes and to evaluate their safety and efficacy in specific cancer types and treatment regimens. Additionally, mechanistic studies are needed to better understand the underlying pathways through which SGLT2 inhibitors exert their cardioprotective and potential anti-tumor effects on cancer therapy-induced cardiotoxicity. As the population of cancer survivors continues to grow, identifying effective strategies to mitigate the cardiovascular complications of cancer treatment becomes increasingly important. SGLT2 inhibitors may represent a promising approach to improve cardiovascular outcomes and overall survival in this vulnerable population.

### Electronic supplementary material

Below is the link to the electronic supplementary material.

**Supplementary Fig. 1.** Funnel plot of all-cause mortality.

**Supplementary Fig. 2** Forest plots of all-cause mortality by cancer types. (a) Breast cancer (b) Lung cancer (c) Hepatoma.

## Data Availability

All data were extracted from publicly available sources and are included in this published article.

## Abbreviations

ARNI: angiotensin receptor-neprilysin inhibitors
CI: confidence interval
DKA: diabetic ketoacidosis
LVEF: left ventricular ejection fraction
NOS: Newcastle-Ottawa Scale
RR: risk ratio
SGLT2: sodium-glucose cotransporter 2
