# Supplementary figures and images for: Cardiovascular outcomes associated with SGLT2 inhibitor therapy in patients with type 2 diabetes mellitus and cancer: a systematic review and meta-analysis
Source: Diabetol Metab Syndr. 2024 May 22;16:108. doi: 10.1186/s13098-024-01354-4 (PMC11110336; doi:10.1186/s13098-024-01354-4)

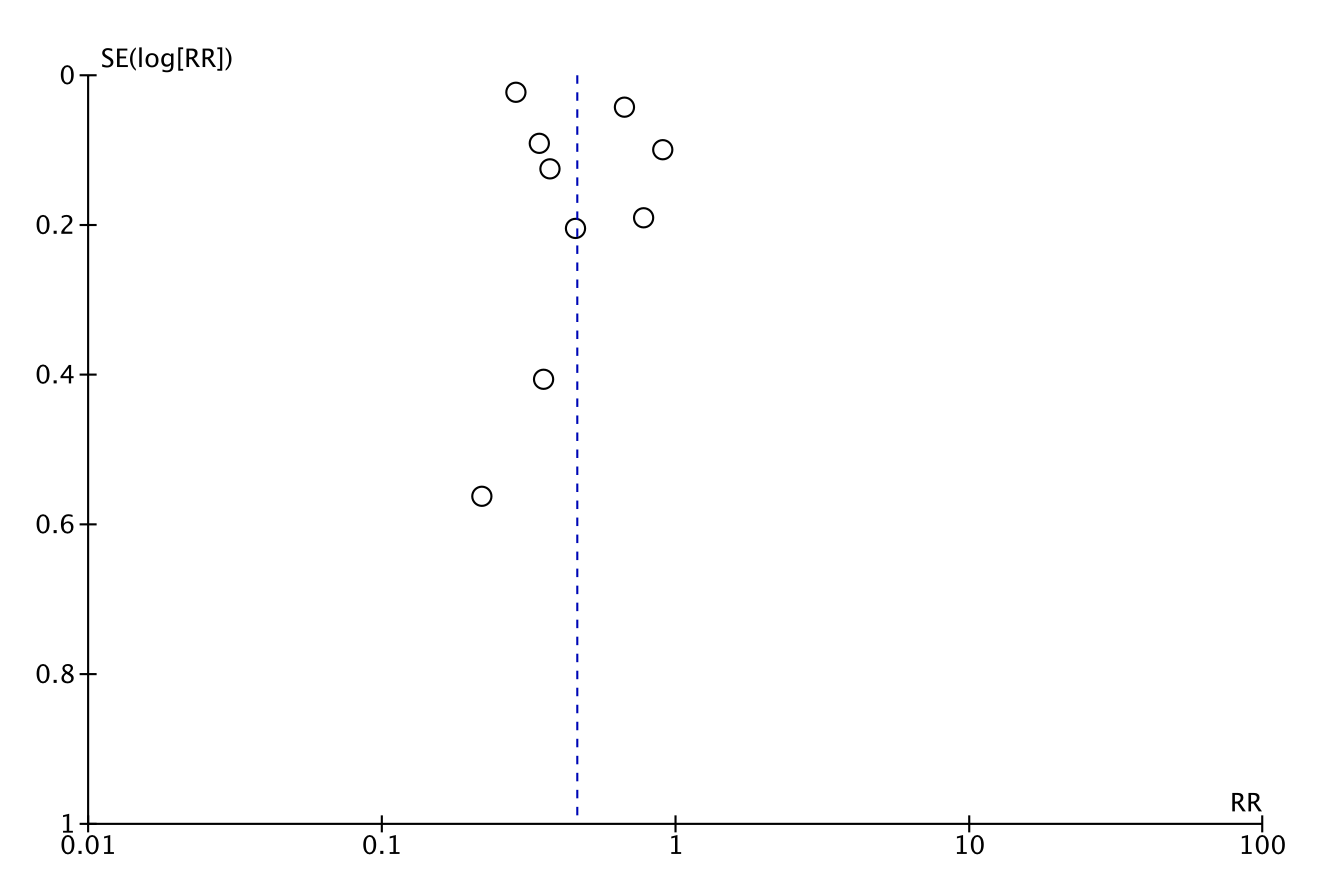

Supplement: Supplementary file 1 — Supplementary Fig. 1. Funnel plot of all-cause mortality. [file 13098_2024_1354_MOESM1_ESM.png]

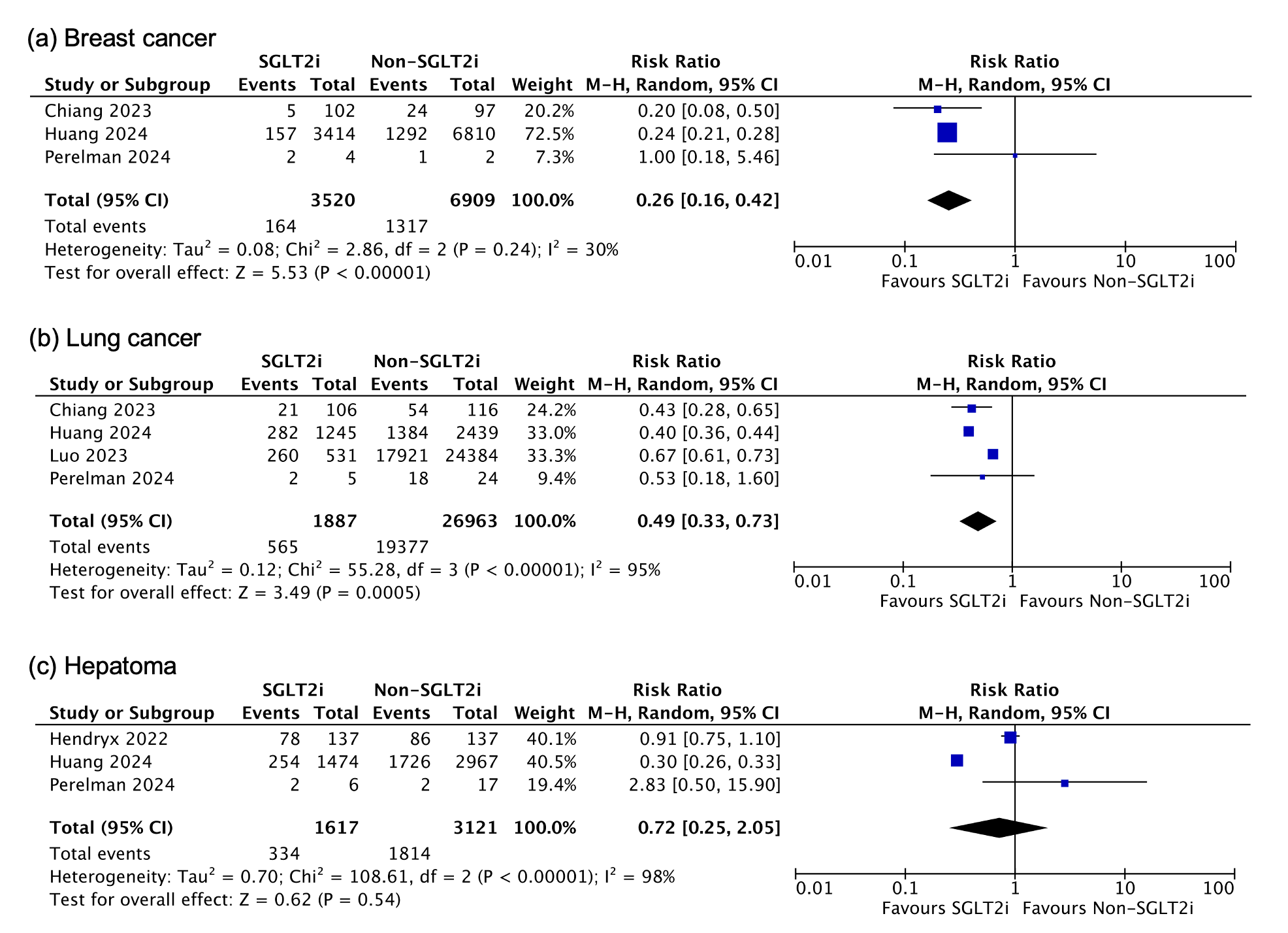

Supplement: Supplementary file 2 — Supplementary Fig. 2 Forest plots of all-cause mortality by cancer types. (a) Breast cancer (b) Lung cancer (c) Hepatoma. [file 13098_2024_1354_MOESM2_ESM.png]
